# Supplementary material for: Frequency-dependent interactions determine outcome of competition between two breast cancer cell lines
Source: Sci Rep. 2021 Mar 1;11:4908. doi: 10.1038/s41598-021-84406-3 (PMC7921689; doi:10.1038/s41598-021-84406-3)
Supplement: Supplementary file 1 — Supplementary Information [file 41598_2021_84406_MOESM1_ESM.pdf]

Supplementary Information: Frequency-dependent interactions determine outcome of competition between two breast cancer cell lines

Audrey R. Freischel[1,2*], Mehdi Damaghi[1*], Jessica J. Cunningham[2], Arig Ibrahim-Hashim[1],Robert J. Gillies[1], Robert A. Gatenby[1,2], Joel S. Brown[1,2]

1 Department of Cancer Physiology, Moffitt Cancer Center, Tampa, FL

2 Department of Integrated Mathematical Oncology, Moffitt Cancer Center, Tampa, FL

*equal contributions

Correspondence:

Experimental: Mehdi.Damaghi@Moffitt.org

Theoretical: Audrey.Freischel@Moffitt.org

MCF-7 Instantaneous Growth Rates					
**Variable**	**Type III Sums of Squares**	**Degrees Freedom**	**Mean Squares**	**F-Ratio**	**P-Value**
**Glucose Concentration (GC)**	18.528	1	18.528	62.389	< 0.00
**pH**	0.014	1	0.014	0.047	0.829
**Starting Frequency (f)**	21.514	4	5.379	18.111	< 0.000
**pH x GC**	5.270	1	5.270	17.747	< 0.000
**f x GC**	0.812	4	0.203	0.684	0.606
**pH x f**	1.755	4	0.439	1.477	0.220
**Error**	18.710	63	0.297		
**MDA-MB-231 Instantaneous Growth Rates**					
**Glucose Concentration (GC)**	0.038	1	0.038	13.957	< 0.00
**pH**	0.172	1	0.172	62.881	< 0.00
**Starting Frequency (f)**	1.157	4	0.289	105.748	< 0.00
**pH x GC**	0.002	1	0.002	0.761	0.386
**f x GC**	0.002	4	0.001	0.224	0.924
**pH x f**	0.011	4	0.003	0.971	0.430
**Error**	0.175	64	0.003		
**Difference Between MCF-7 and MDA-MB-231 Growth Rates**					
**Glucose Concentration (GC)**	19.473	1	19.473	57.178	< 0.00
**pH**	0.013	1	0.013	0.038	0.846
**Starting Frequency (f)**	1.680	3	0.560	1.644	0.191
**pH x GC**	4.896	1	4.896	14.377	< 0.000
**f x GC**	0.281	3	0.094	0.275	0.843
**pH x f**	0.999	3	0.333	0.977	0.411
**Error**	17.369	51	0.341		

Supplementary table 1: ANOVA results to determine statistical significance of culture conditions on growth rate for experiment with high seeding density (20,000 cells) and glucose concentrations of 0 g/L and 2 g/L.

MCF-7 Instantaneous Growth Rates					
**Variable**	**Type III Sums of Squares**	**Degrees Freedom**	**Mean Squares**	**F-Ratio**	**P-Value**
**Glucose Concentration (GC)**	0.018	1	0.018	0.138	0.711
**pH**	1.126	1	1.126	8.852	0.004
**Starting Frequency (f)**	26.713	4	6.678	52.525	< 0.000
**pH x GC**	0.953	1	0.953	7.499	0.008
**f x GC**	0.298	4	0.075	0.586	0.674
**pH x f**	1.307	4	0.327	2.570	0.046
**Error**	8.137	64	0.127		
**MDA-MB-231 Instantaneous Growth Rates**					
**Glucose Concentration (GC)**	0.009	1	0.009	4.547	0.037
**pH**	0.111	1	0.111	53.146	< 0.000
**Starting Frequency (f)**	0.964	4	0.241	115.872	< 0.000
**pH x GC**	0.006	1	0.006	2.727	0.104
**f x GC**	0.009	4	0.002	1.115	0.357
**pH x f**	0.041	4	0.010	4.987	0.001
**Error**	0.133	64	0.002		
**Difference Between MCF-7 and MDA-MB-231 Growth Rates**					
**Glucose Concentration (GC)**	0.004	1	0.004	0.024	0.877
**pH**	1.392	1	1.392	9.390	0.003
**Starting Frequency (f)**	10.427	3	3.476	23.445	< 0.000
**pH x GC**	1.049	1	1.049	7.075	0.010
**f x GC**	0.186	3	1.049	0.419	0.740
**pH x f**	1.236	3	0.412	2.778	0.050
**Error**	7.561	51	0.148		

Supplementary table 2: ANOVA results to determine statistical significance of culture conditions on growth rate for experiment with high seeding density (20,000 cells) and glucose concentrations of 1 g/L and 4.5 g/L.

MCF-7 Instantaneous Growth Rates +glutamine					
**Variable**	**Type III Sums of Squares**	**Degrees Freedom**	**Mean Squares**	**F-Ratio**	**P-Value**
**Glucose Concentration (GC)**	4.955	4	1.239	3.640	0.011
**pH**	0.538	1	0.538	1.580	0.214
**Starting Frequency (f)**	0.216	1	0.216	0.636	0.429
**pH x GC**	1.504	4	0.376	1.104	0.364
**f x GC**	0.784	4	0.196	0.576	0.681
**pH x f**	0.443	1	0.443	1.301	0.259
**Error**	18.721	55	0.340		
**MDA-MB-231 Instantaneous Growth Rates +glutamine**					
**Glucose Concentration (GC)**	0.074	1	0.074	4.283	0.043
**pH**	0.127	1	0.127	7.318	0.009
**Starting Frequency (f)**	0.904	4	0.226	13.004	< 0.000
**pH x GC**	0.006	1	0.006	0.367	0.547
**f x GC**	0.014	4	0.004	0.204	0.935
**pH x f**	0.116	4	0.029	1.672	0.167
**Error**	1.113	64	0.017		
**MCF-7 Instantaneous Growth Rates -glutamine**					
**Variable**	**Type III Sums of Squares**	**Degrees Freedom**	**Mean Squares**	**F-Ratio**	**P-Value**
**Glucose Concentration (GC)**	1.655	1	1.655	3.177	0.080
**pH**	5.023	1	5.023	9.641	0.003
**Starting Frequency (f)**	12.791	4	3.198	6.138	< 0.000
**pH x GC**	2.309	1	2.309	4.432	0.039
**f x GC**	4.895	4	1.224	2.349	0.064
**pH x f**	7.648	4	1.912	3.670	0.010
**Error**	31.262	60	0.521		
**MDA-MB-231 Instantaneous Growth Rates -glutamine**					
**Glucose Concentration (GC)**	0.016	1	0.016	1.116	0.295
**pH**	0.113	1	0.113	7.968	0.006
**Starting Frequency (f)**	0.930	4	0.233	16.358	< 0.000
**pH x GC**	0.026	1	0.026	1.794	0.185
**f x GC**	0.098	4	0.025	1.731	0.154
**pH x f**	0.146	4	0.036	2.560	0.047
**Error**	0.910	64	0.014		

Supplementary table 3: ANOVA results to determine statistical significance of culture conditions on growth rate for experiment with low seeding density (10,000 cells) and glucose concentrations of 0 g/L and 4.5 g/L.

MCF-7 Carrying Capacity					
**Variable**	**Type III Sums of Squares**	**Degrees Freedom**	**Mean Squares**	**F-Ratio**	**P-Value**
**Glucose Concentration (GC)**	1.503	1	1.503	0.008	0.930
**pH**	195.016	1	195.016	1.047	0.328
**pH x GC**	1,384.361	1	1,384.361	7.435	0.020
**Error**	2,048.017	11	186.183		
**MDA-MB-231 Carrying Capacity**					
**Glucose Concentration (GC)**	1,116.013	1	1,116.013	610.634	< 0.00
**pH**	1,237.514	1	1,237.514	677.114	< 0.00
**pH x GC**	972.488	1	972.488	532.103	< 0.000
**Error**	116.968	64	1.828		

MCF-7 Carrying Capacity					
**Variable**	**Type III Sums of Squares**	**Degrees Freedom**	**Mean Squares**	**F-Ratio**	**P-Value**
**Glucose Concentration (GC)**	18.431	1	18.431	0.400	0.539
**pH**	593.962	1	593.962	12.875	0.004
**pH x GC**	398.561	1	398.561	8.639	0.012
**Error**	553.593	12	46.133		
**MDA-MB-231 Carrying Capacity**					
**Glucose Concentration (GC)**	181.817	1	181.817	67.181	< 0.000
**pH**	66.078	1	66.078	24.416	< 0.000
**pH x GC**	71.761	1	71.761	26.516	< 0.000
**Error**	205.683	76	2.706		

Supplementary table 4: ANOVA results to determine statistical significance of culture conditions on carrying capacity for experiment with high seeding density (20,000 cells) and glucose concentrations of 0 g/L and 2 g/L in top table and 1 g/L and 4.5 g/L in lower table.

MCF-7 Carrying Capacity +glutamine					
Variable	Type III Sums of Squares	Degrees Freedom	Mean Squares	F-Ratio	P-Value
Glucose Concentration (GC)	371.403	1	371.403	8.314	0.005
pH	270.455	1	270.455	6.054	0.017
Starting Frequency (f)	8,201.115	4	2,050.279	45.896	< 0.000
pH x GC	0.109	1	0.109	0.002	0.961
f x GC	481.061	4	120.265	2.692	0.039
pH x f	172.672	4	43.168	0.966	0.432
Error	2,858.997	64	44.672		
MDA-MB-231 Carrying Capacity + glutamine					
Glucose Concentration (GC)	0.856	1	0.856	1.784	0.186
pH	125.666	1	125.666	261.952	< 0.000
Starting Frequency (f)	220.961	4	55.240	115.149	< 0.000
pH x GC	1.260	1	1.260	2.626	0.110
f x GC	7.240	4	1.810	3.773	0.008
pH x f	57.634	4	14.408	30.034	< 0.000
Error	30.703	64	0.480		

MCF-7 Carrying Capacity -glutamine					
Variable	Type III Sums of Squares	Degrees Freedom	Mean Squares	F-Ratio	P-Value
Glucose Concentration (GC)	1,419,397.848	1	1,419,397.848	3.890	0.053
pH	1,941,483.025	1	1,941,483.025	5.321	0.024
Starting Frequency (f)	1.726E+008	4	43,154,454.625	118.278	< 0.000
pH x GC	1,291,221.858	1	1,291,221.858	3.539	0.064
f x GC	686,257.624	4	171,564.406	0.470	0.757
pH x f	16,814,748.188	4	4,203,687.047	11.521	< 0.000
Error	23,350,855.237	64	364,857.113		
MDA-MB-231 Carrying Capacity -glutamine					
Glucose Concentration (GC)	47,425.034	1	47,425.034	7.808	0.007
pH	22,960.408	1	22,960.408	3.780	0.056
Starting Frequency (f)	1,143,424.578	4	285,856.144	47.060	< 0.000
pH x GC	78,851.808	1	78,851.808	12.981	0.001
f x GC	156,395.360	4	39,098.840	6.437	< 0.000
pH x f	26,175.583	4	6,543.896	1.077	0.375
Error	388,752.068	64	6,074.251		

Supplementary table 5: ANOVA results to determine statistical significance of culture conditions on carrying capacity for experiment with low seeding density (10,000 cells) and glucose concentrations of 0 g/L and 4.5 g/L.

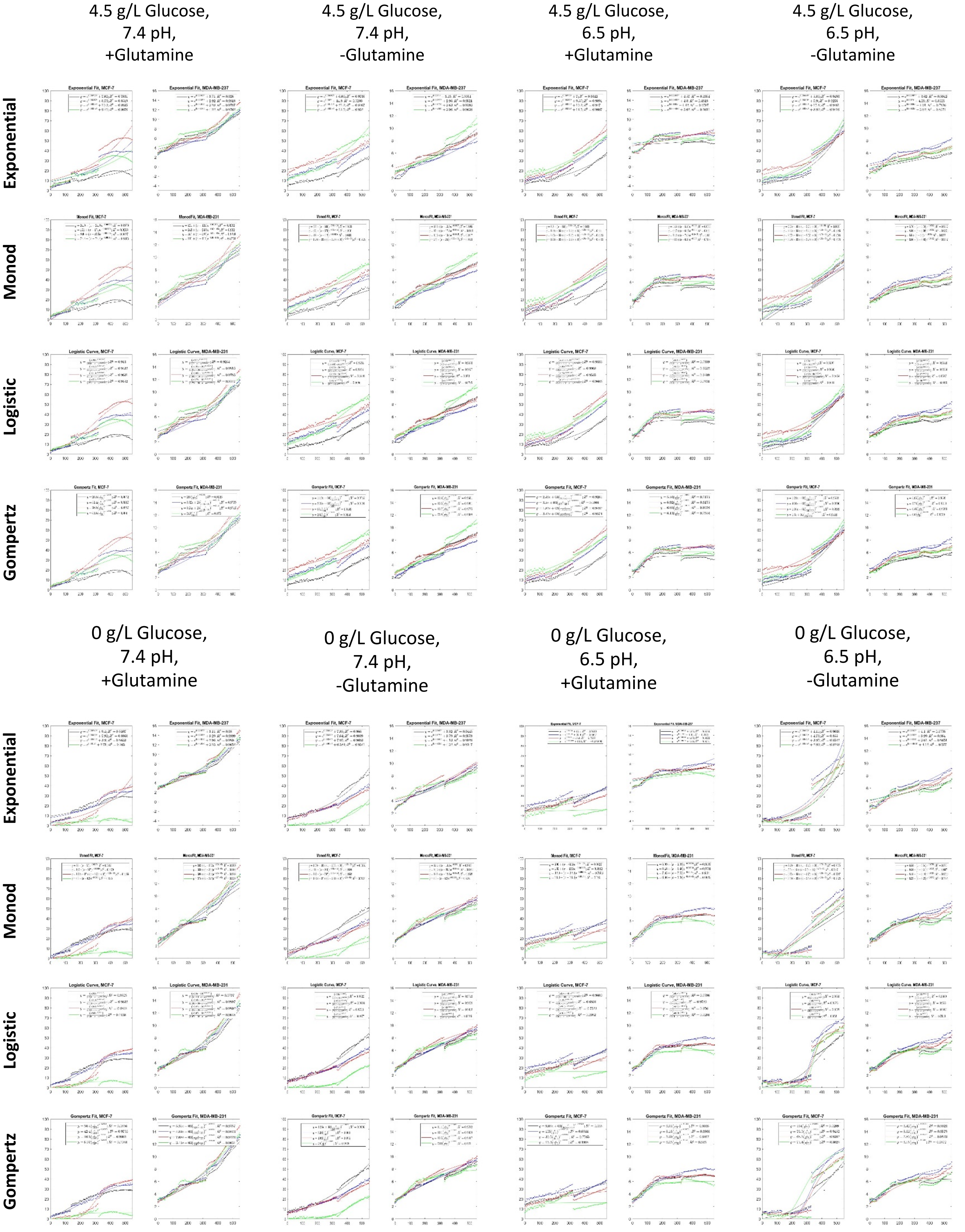

Supplementary Figure 1: Fits of the exponential, Monod-like, logistic and Gompertz growth models to monoculture spheroid data. Fitting was performed using nonlinear constrained optimization.

Adjusted R^2					
**Variable**	**Type III Sums of Squares**	**Degrees Freedom**	**Mean Squares**	**F-Ratio**	**P-Value**
**Model**	0.430	3	0.143	9.966	< 0.000
**Culture Condition**	1.013	7	0.145	10.074	< 0.000
**Cell Type**	0.025	1	0.025	1.726	0.190
**Culture Condition x Model**	0.272	21	0.013	0.901	0.589
**Cell Type x Model**	0.154	3	0.051	3.568	0.015
**Cell Type x Culture Condition**	0.990	7	0.141	9.845	< 0.000
**Error**	3.060	213	0.014		
**RMSE**					
**Model**	280.951	3	93.650	5.894	0.001
**Culture Condition**	2,670.428	7	381.490	24.009	< 0.000
**Cell Type**	17,782.592	1	17,782.592	1,119.135	< 0.000
**Culture Condition x Model**	57.078	21	2.718	0.171	1.000
**Cell Type x Model**	127.607	3	42.536	2.677	0.048
**Cell Type x Culture Condition**	3,175.509	7	453.644	28.550	< 0.000
**Error**	3,384.481	213	15.890		

Supplementary table 6: Results of ANOVA comparing quality of fit measurements (Adjusted R^2 and RMSE) for all four growth models.

MCF-7 Slope of Carrying Capacity +glutamine					
**Variable**	**Type III Sums of Squares**	**Degrees Freedom**	**Mean Squares**	**F-Ratio**	**P-Value**
**Glucose Concentration (GC)**	0.000	1	0.000	0.099	0.754
**pH**	0.030	1	0.030	86.905	< 0.000
**Starting Frequency (f)**	0.038	4	0.009	27.563	< 0.000
**pH x GC**	0.003	1	0.003	7.412	0.008
**f x GC**	0.000	4	0.000	0.298	0.878
**pH x f**	0.026	4	0.006	18.994	< 0.000
**Error**	0.022	64	0.000		
**MDA-MB-231 Slope of Carrying Capacity +glutamine**					
**Glucose Concentration (GC)**	0.000	1	0.000	16.406	< 0.000
**pH**	0.003	1	0.003	358.724	< 0.000
**Starting Frequency (f)**	0.001	4	0.000	19.839	< 0.000
**pH x GC**	0.000	1	0.000	1.528	0.221
**f x GC**	0.000	4	0.000	1.084	0.372
**pH x f**	0.002	4	0.000	56.042	< 0.000
**Error**	0.000	64	0.000		
**MCF-7 Slope of Carrying Capacity -glutamine**					
**Variable**	**Type III Sums of Squares**	**Degrees Freedom**	**Mean Squares**	**F-Ratio**	**P-Value**
**Glucose Concentration (GC)**	1.019	1	1.019	0.837	0.364
**pH**	3.316	1	3.316	2.723	0.104
**Starting Frequency (f)**	1,287.534	4	321.884	264.291	< 0.000
**pH x GC**	0.223	1	0.223	0.183	0.670
**f x GC**	10.263	4	2.566	2.107	0.090
**pH x f**	100.048	4	25.012	20.537	< 0.000
**Error**	77.946	64	1.218		
**MDA-MB-231 Slope of Carrying Capacity −glutamine**					
**Glucose Concentration (GC)**	0.618	1	0.618	9.652	0.003
**pH**	0.139	1	0.139	2.179	0.145
**Starting Frequency (f)**	1.793	4	0.448	7.005	< 0.000
**pH x GC**	1.151	1	1.151	17.982	< 0.000
**f x GC**	0.234	4	0.058	0.912	0.462
**pH x f**	1.646	4	0.412	6.430	< 0.000
**Error**	4.096	64	0.064		

Supplementary table 7: ANOVA comparing the final slopes of the low seeding density spheroids. Carrying capacity values were determined using the monoculture spheroids for MCF-7 and all spheroids for MDA-MB-231.
